# Non-Destructive Assessment of Beef Freshness Using Visible and Near-Infrared Spectroscopy with Interpretable Machine Learning

**DOI:** 10.3390/foods15040728

**Published:** 2026-02-15

**Authors:** Ruoxin Chen, Wei Ning, Xufen Xie, Jingran Bi, Gongliang Zhang, Hongman Hou

**Affiliations:** 1 College of Food Science and Technology, Dalian Polytechnic University, Dalian 116034, China; 2 School of Information Science and Engineering, Dalian Polytechnic University, Dalian 116034, China

**Keywords:** beef freshness, Vis-NIR spectroscopy, feature selection, PSOGA, SHAP

## Abstract

Beef freshness is a critical indicator of meat quality and safety, and its rapid, non-destructive detection is of significant importance for ensuring consumer health and enhancing quality control throughout the meat industry chain. This study developed a novel methodology for non-destructive beef freshness assessment using visible and near-infrared (Vis-NIR) spectroscopy combined with machine learning, explainable artificial intelligence (xAI) techniques, and the SHapley Additive exPlanations (SHAP) framework. An improved hybrid heuristic method, particle swarm optimization–genetic algorithm (PSOGA), was used for feature selection, optimizing the wavelength subset for predicting beef quality indicators, including total volatile basic nitrogen (TVB-N) and color parameters (L*, a*, and b*). The eXtreme Gradient Boosting (XGBoost) was employed for regression modeling, and the results showed that PSOGA significantly outperforms traditional methods, with the PSOGA-XGBoost model achieving a satisfactory prediction accuracy (R^2^p values of 0.9504 for TVB-N, 0.9540 for L*, 0.8939 for a*, and 0.9416 for b*). The SHAP framework identified the key wavelengths as 1236 nm and 1316 nm for TVB-N, 728 nm for L*, 576 nm for a*, and 604 nm for b*, providing valuable insights into the determination of key wavelengths and enhancing the interpretability of the model. The results demonstrated the effectiveness of PSOGA and SHAP, providing a promising analytical method for monitoring beef freshness.

## 1. Introduction

In an era of escalating global high-quality protein demand, beef, prized for its nutrients and flavor [1], confronts a formidable hurdle in storage, transportation, and distribution due to its perishability, impacting food safety and consumer trust. The deterioration of beef quality is primarily a consequence of microbial proliferation and enzymatic reactions, with total volatile basic nitrogen (TVB-N) serving as a critical indicator of spoilage [2]. As spoilage progresses, TVB-N levels increase significantly, reflecting the accumulation of protein degradation products, and thus providing a reliable biochemical marker for freshness assessment. Concurrently, changes in meat color (L*, a*, b*) are pivotal visual cues guiding consumer purchasing decisions and are tightly linked to myoglobin oxidation and other storage-dependent biochemical changes [3]. Although conventional analytical assays quantify these indicators with precision, they are invasive, destructive, and time-consuming, limiting their suitability for rapid, real-time, and non-destructive quality control [4].

In recent years, visible–near-infrared (Vis-NIR) spectroscopy has become a mainstream technique for food quality analysis by virtue of its non-destructive, high-throughput, and multi-parameter detection advantages [5,6,7]. Despite the significant potential of Vis-NIR spectroscopy technology, the complexity of its high-dimensional spectral data [8] may still weaken the generalization ability of the model. Effective wavelength selection is therefore pivotal.

Recent meta-heuristic feature selection methods mitigate some limitations of classical approaches in high-dimensional spaces but can suffer from slow convergence and local optima [9,10]. Hybrid strategies that pair global search with local refinement have shown improved stability and performance across diverse datasets [11]. However, existing hybrid methods often struggle to balance convergence speed with population diversity, leading to premature stagnation. Furthermore, in many practical settings, it is not enough to achieve accuracy; model transparency is equally important. Most current spectroscopic models function as “black boxes,” lacking the interpretability required to link spectral features to underlying biochemical mechanisms. Explainable artificial intelligence (xAI) methods, particularly SHapley Additive exPlanations (SHAP), quantify each feature’s contribution to predictions with a principled game-theoretic basis, making them well-suited to interpret spectroscopic models and to highlight truly informative wavelengths [12,13].

This study aims to address these limitations by integrating an improved hybrid heuristic—the particle swarm optimization–genetic algorithm (PSOGA)—with the SHAP framework for beef freshness assessment. The novelty lies in the synergistic combination of PSOGA’s advanced feature selection capabilities (enhanced by logistic mapping and Lévy flight mutation) and interpretability of SHAP, enabling both high-precision prediction and biochemical validation. We use eXtreme Gradient Boosting (XGBoost), a gradient-boosting framework adept at modeling nonlinear relationships in high-dimensional data, to predict TVB-N and color parameters (L*, a*, b*) from Vis-NIR spectra [14].

The specific objectives of this study are: (i) to develop an XGBoost model suitable for beef freshness indicators (TVB-N and L*, a*, b*) using visible–near-infrared spectroscopy; (ii) to optimize the model by screening dominant wavelengths using the proposed PSOGA and compare its performance with four other feature selection methods; and (iii) to explain the contribution of each input feature to the model prediction using SHAP to comprehensively understand the screening mechanism of feature wavelengths in Vis-NIR spectroscopy.

## 2. Materials and Methods

### 2.1. The Sample Collection

The samples used in the experiments were purchased from the meat counter of local Qianhe Market (Dalian, China) within 24 h of slaughter and were quickly transported to the laboratory within 30 min. To ensure sufficient biological variability and model robustness, the samples were collected from multiple different cattle carcasses rather than a single animal. This sampling approach accounts for individual differences in meat composition and initial freshness, thereby improving the generalization capability of the subsequent prediction models. In the laboratory, the beef loins were cut into uniform 6 cm × 6 cm × 2 cm slices on an aseptic table, and a total of 420 samples were taken, and the surface fat and sinew were removed to ensure a uniform and consistent sample surface. Each beef sample was then individually packaged in a sealed plastic bag before being placed in an environmental incubator. The dataset used to predict beef freshness indicators (TVB-N and L*, a*, b*) contained the variation in beef samples under different storage conditions. 240 samples were stored at 4 °C and sampled on days 0, 1, 3, 5, 7, 8, 9 and 10 of storage, with 30 samples examined at a time, while 180 samples were stored at 25 °C at room temperature and were sampled on days 0, 1, 2, and 3 of storage, with 45 samples examined at a time. The sample distribution (240 samples at 4 °C and 180 samples at 25 °C) was determined based on the distinct spoilage rates of meat at different temperatures. Since the biochemical degradation of beef at 4 °C is significantly slower than at 25 °C, a longer sampling period and more frequent sampling points were necessary for the refrigerated group to capture the gradual evolution of freshness indicators with sufficient temporal resolution. Conversely, the rapid spoilage at 25 °C allowed for a shorter experimental duration. This strategy ensured that the dataset contained a balanced representation of “fresh,” “semi-fresh,” and “spoiled” states for both temperature settings, providing a robust foundation for model training. The samples were scanned in the Vis-NIR spectra, and the TVB-N content and the colorimetric parameters L*, a*, and b* were measured during each test.

### 2.2. Measurement of Vis-NIR Spectra

Spectral data for beef samples were collected in reflectance mode using a UV/Vis/NIR spectrophotometer (Lambda 950, PerkinElmer, Shelton, CT, USA) equipped with deuterium and tungsten halogen light sources. Prior to acquisition, the instrument was warmed up for 30 min to stabilize the light source and minimize drift. To ensure data stability and minimize the influence of potential instrument drift, background correction was performed using a standard white reference every 30 min during the measurement process. The spectra were collected from 400 to 1800 nm with a resolution of 4 nm and contained a total of 351 variables.

The beef samples were removed from the environmental incubator and equilibrated at a laboratory temperature of approximately 25 °C for 30 min to ensure that the samples reached room temperature. Subsequently, surface moisture and exudate generated during storage were gently removed using filter paper. In order to obtain the Vis-NIR spectra of each sample, three different points on the beef surface were randomly selected and scanned, and the resulting spectra were averaged for subsequent analysis. This treatment not only ensures the consistency of the sample state, but also improves the reliability and accuracy of the data through multiple measurements. In addition, the use of averaged spectra can reduce the influence of local variability on the overall results, thus providing a more robust data base for subsequent model construction.

### 2.3. Measurement of Reference Values

#### 2.3.1. L*, a*, b* Values

A precision colorimeter (DS-700D, Hangzhou Color Spectrum Technology Co., Ltd. Hangzhou, China) was used to determine the L*, a*, and b* values of beef samples. The colorimeter was set to L*, a*, and b*, with a light source of D65, a standard observer angle of 10°, an aperture size of 11 nm, and a measurement time of 1 s. Prior to the measurements, the bottom plate of the colorimeter was rinsed with distilled water and wiped dry with a wipe paper, and then calibrated using the white and black references in turn. The L*, a* and b* values were measured at three different locations on the surface of the beef sample, and the average of the three times was used as the final color reference value of the sample. The repeatability of the colorimeter measurements was high, with a relative standard deviation (RSD) of less than 2% across triplicate readings. During the measurement process, it was important to avoid the fat and connective tissues of the beef and to keep the measurement holes close to the sample under test to prevent light leakage from affecting the accuracy of the data.

#### 2.3.2. TVB-N Values

The content of TVB-N was determined using the semi-micro Kjeldahl method according to the Chinese National Food Safety Standard GB 5009.228-2016 [15]. A total of 10 g of beef sample and 75 mL of water were mixed in a distillation tube, shaken well, and immersed for 30 min; then, 1 g of MgO was added and distilled for 3 min. At the end of the reaction, a mixing indicator was added to the distillate. The samples were calibrated with standard acid, and the content of TVB-N was calculated. Three measurements were taken simultaneously for each sample, and the average value was recorded. The method demonstrated excellent precision with an RSD of <5%, and the detection limit (LOD) was approximately 0.1 mg/100 g, ensuring accurate quantification across the full range of freshness levels.

### 2.4. Spectral Pre-Processing

The raw spectral signals can be disturbed due to environmental changes, electronic noise and sample scattering. To enhance model robustness, five common pre-processing techniques targeting different noise sources were compared: multiplicative scatter correction (MSC) and standard normal variate (SNV) for correcting scatter and path length variations [16,17]; Savitzky–Golay (SG) smoothing for noise reduction [18]; and SG first-order (SG + 1D) and second-order (SG + 2D) derivatives for resolving overlapping peaks and enhancing spectral features [19].

The selection of the optimal pre-processing method for each target variable (TVB-N and color parameters L*, a* and b*) was determined empirically based on model performance. Specifically, XGBoost models were built using both raw and pre-processed spectra, and the method yielding the lowest root mean squared error of prediction (RMSEP) and highest coefficient of determination (R^2^p) was selected for subsequent feature selection and final model development. This data-driven approach ensures that the chosen pre-processing maximizes the signal-to-noise ratio specific to each freshness indicator.

### 2.5. PSOGA Feature Selection

This study proposed an improved hybrid algorithm-PSOGA. The PSO algorithm was used to maintain high convergence efficiency and accuracy, while GA algorithm was used to avoid getting stuck in local optima. By combining and improving these two algorithms, the selection of characteristic wavelengths was achieved [20,21]. XGBoost model and 3-fold cross-validation were used to calculate the fitness function (mean squared error, MSE) and evaluate the performance of the subset. The improvements to PSOGA primarily included the introduction of a logical mapping algorithm in the PSO component to initialize the population and enhance population diversity [22,23]. Second, the population was divided into elite subpopulations and ordinary subpopulations using adaptive classification coefficients, and a nonlinear decreasing inertia weight was introduced to balance local exploitation and global search capabilities [24,25]. Lévy flight was integrated into the mutation stage of the genetic algorithm [26], and polynomial mutation was adopted to enhance population diversity. The detailed mathematical formulations and specific algorithmic improvements are provided in the Supplementary Materials (Sections S1–S3, Equations (S1)–(S8)).

The workflow of PSOGA comprises three main stages: (1) Initialization: The population is distributed uniformly across the search space using logistic mapping. (2) Hybrid Optimization: In each iteration, particles are categorized into elite and ordinary groups based on fitness. Elite particles update positions via an improved non-linear inertia weight strategy to refine local search, while ordinary particles undergo genetic crossover and mutation to maintain population diversity. (3) Selection: The process iterates until the termination criterion is met, selecting the feature subset that minimizes the RMSE.

To ensure a fair comparison, the population size (N=50) and maximum iterations (T=100) were consistent across all algorithms (PSOGA, PSO, GA, GWO). The computational cost was evaluated on a standard laptop computer (AMD Ryzen 7 3700U CPU @ 2.30 GHz, 8 GB RAM). The average execution time for selecting the optimal wavelength subset was approximately 15 min per indicator. It is important to note that this computational expense is limited to the offline training phase; once the dominant wavelengths are identified, the final model provides rapid, near-real-time predictions (<0.1 s per sample), fulfilling the requirements for industrial application.

### 2.6. Model Establishment

XGBoost was employed to construct the regression models for TVB-N and color parameters due to its superior ability to capture non-linear relationships and its inherent regularization mechanisms that prevent overfitting [27]. Instead of relying on default settings, key hyperparameters—including the number of estimators, learning rate, and maximum depth—were optimized using a grid search strategy to ensure model robustness. The specific hyperparameter ranges and boundary conditions are summarized in Table 1. This approach balances the learning capacity of the model with its generalization performance on high-dimensional spectral datasets.

### 2.7. SHAP Model

This study employed SHAP to interpret the most accurate predictive models for beef TVB-N and L*, a*, b*. The SHAP analysis was performed using the TreeExplainer algorithm, which is optimized for tree-based machine learning models. To evaluate the decision-making process of the model on unseen data, SHAP values were computed specifically on the prediction set. Thanks to the efficiency of TreeExplainer, the computational overhead was minimal (approximately 5–10 s per indicator). The interpretation focused on the specific subset of characteristic wavelengths selected by the PSOGA, quantifying the contribution of each selected feature to the final freshness prediction.

### 2.8. Evaluation of Models

**Figure 1:**
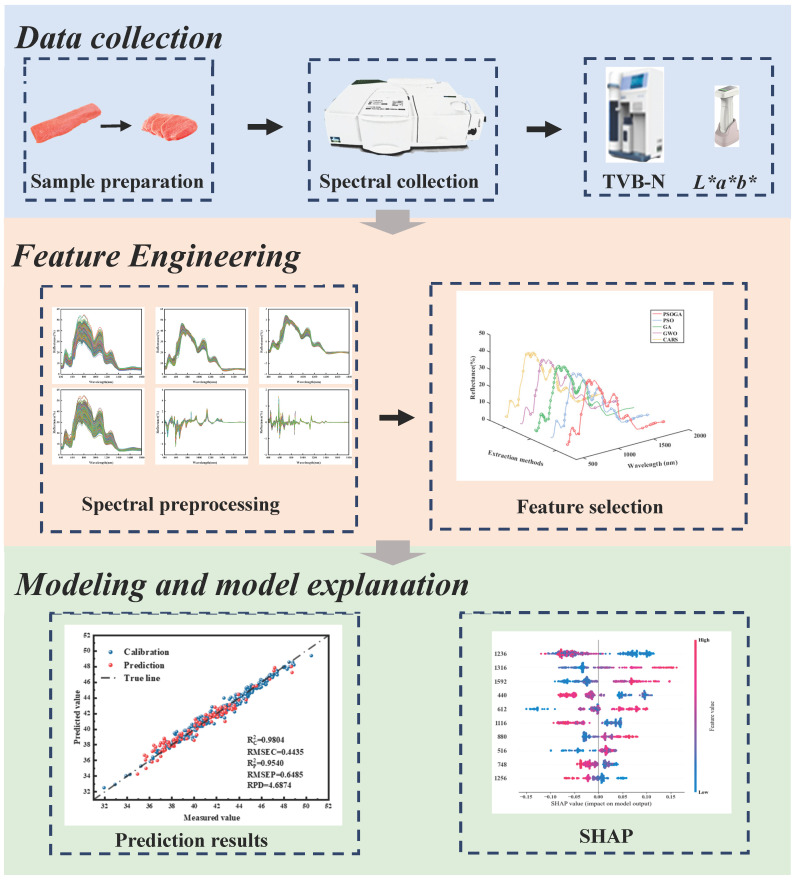
The regression analytical framework.

In this study, the coefficient of determination of the correction set (R^2^c), the coefficient of determination of the prediction set (R^2^p), the root mean square error of the correction set (RMSEC) and the root mean square error of the prediction set (RMSEP), and the relative prediction deviation (RPD) were used to evaluate the performance of the model. In general, the model performance was categorized as excellent only when it demonstrated both a high R^2^p and a minimal discrepancy between RMSEC and RMSEP, rather than depending on RPD thresholds alone. All code was written and tested using Python in the PyCharm 2020.1.3 integrated development environment. The system environment was configured using computational libraries such as NumPy, Pandas, and Scikit-learn. The key steps of the experiment are shown in Figure 1.

## 3. Results and Discussions

### 3.1. Experimental Data and Spectral Analysis

In this study, the TVB-N content and L*a*b* of beef tenderloin during storage were analyzed. Sample set partitioning based on the joint x-y distance (SPXY) algorithm [28] was used to divide the data into a calibration set consisting of 315 samples and a prediction set consisting of 105 samples with a ratio of 3:1. As shown in Table 2, the statistical metrics of the calibration and prediction datasets are listed, demonstrating the range and variability of each metric. It can be seen that there is a large range of variability in these metrics due to the different storage times of the beef samples, and a large range of variability facilitates robust modeling. The calibration and prediction data have a uniform sample distribution. The calibration data has a wider range than the predicted data, which helps to avoid bias between the two distributions. In this study, beef samples were preserved at different temperatures to ensure that the measurement metrics covered a wider range, thus enhancing the robustness of the predictive model [29]. This approach supports the development of more general and reliable predictive models.

**Figure 2:**
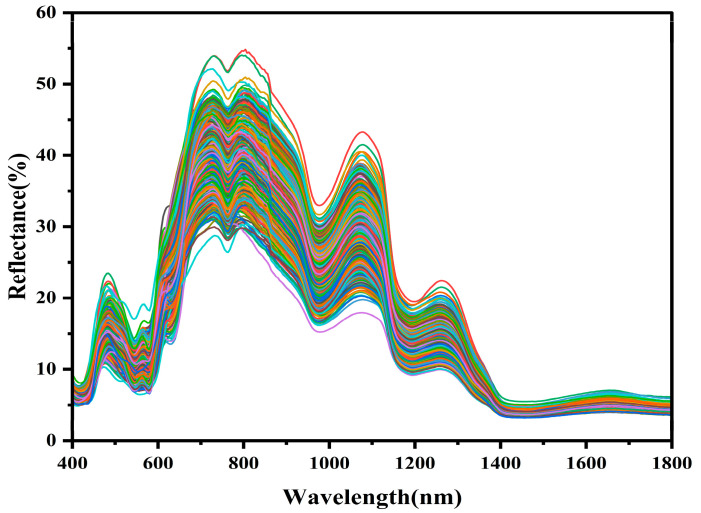
Original Vis-NIR spectra of beef samples.

Figure 2 presents the raw Vis-NIR spectra of the samples. It can be seen that the spectral trends of all samples are generally consistent, although there are some differences in reflectance intensity. These differences in intensity are attributed to changes in the chemical composition of the beef during storage, which affects the reflectance of the samples. This observation further demonstrates that the spectra contain a great deal of information about the samples, with absorption wavelengths at 415 nm and 470 nm associated primarily with myoglobin [30]. A distinct absorption band exists in the visible region near 580 nm, which mainly corresponds to the absorption properties of oxy-myoglobin, myoglobin, and deoxy-myoglobin [31]. In addition, absorption peaks near 550 nm, 760 nm, 980 nm, 1180 nm, and 1450 nm are also identified as closely related to deoxy-myoglobin and oxy-myoglobin [32]. In the near-infrared region, the absorption near 750 nm is thought to be related to the triplet state jump of the NH_3_^+^ group and the third overtone of O–H. The absorption at 1080 nm corresponds to the second overtone of N–H. In addition, the absorption at around 1200 nm corresponds to the second overtone of N–H. The absorption at around 1200 nm corresponds to the second overtone of C–H [33]. The absorption at 1450 nm is associated with the first overtones of O–H and N–H. The results are consistent with previous findings in meat quality spectral analysis and further validate the importance of this band in the prediction of beef freshness.

### 3.2. Spectral Pre-Processing and XGBoost Models Using the Full Set of Wavelengths

**Figure 3:**
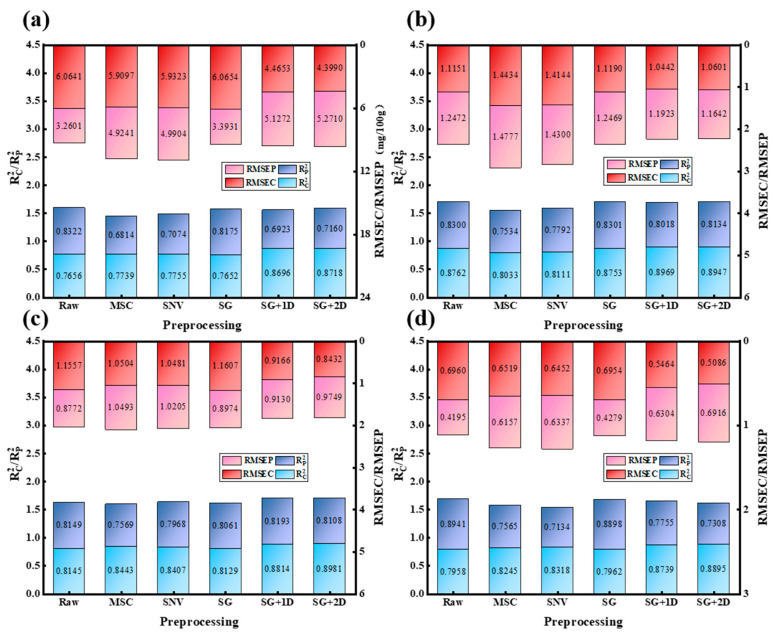
Comparison of statistical results of pre-processed sample sets: (**a**) TVB-N content, (**b**) L*, (**c**) a*, (**d**) b*.

In order to predict the TVB-N content and L*a*b* value of beef samples, full-spectrum-based XGBoost models were built using raw spectra and spectra pre-processed by five methods, namely, MSC, SNV, SG, SG + 1D, and SG + 2D, respectively, and the prediction performances of these models were evaluated, and the results are shown in Figure 3. For TVB-N and b*, the models based on raw spectra achieved the highest accuracy. This suggests that the original signals contained essential chemical information and that inappropriate pre-processing could lead to information loss [34]. Consequently, raw spectra were retained for these indicators. Regarding L*, SG smoothing yielded results statistically comparable to the raw spectra. Although the numerical difference in RMSEP was negligible, SG was selected to enhance model robustness by mitigating potential random noise fluctuations, thereby minimizing the risk of overfitting [35]. Similarly, for a*, SG + 1D pre-processing provided a slight improvement in performance (R^2^p increased from 0.815 to 0.819). This indicates that the first-order derivative effectively highlighted spectral features related to redness while suppressing baseline drift [36]. Based on these empirical comparisons, the optimal pre-processing strategy was individually determined for each target variable to balance accuracy and stability.

### 3.3. Feature Wavelengths Selection and XGBoost Models at Selected Wavelengths

The Vis-NIR spectra (400–1800 nm) collected in this study totaled 351 data points. However, modeling does not always require high-resolution data spectra, and the results of data that have been preprocessed but not feature-selected are slightly improved but still unsatisfactory, and appropriate feature extraction strategies can also improve the prediction accuracy of the model [37]. In order to comprehensively evaluate the effectiveness of different feature selection methods, we analyzed the proposed PSOGA in comparison with three other evolutionary algorithms: PSO, GA, and gray wolf optimization algorithm (GWO). In addition, competitive adaptive re-weighted sampling (CARS) methods based on weighted sampling were examined to evaluate their performance in screening key feature wavelengths.

#### 3.3.1. Distribution of Feature Wavelengths by Different Methods

**Figure 4:**
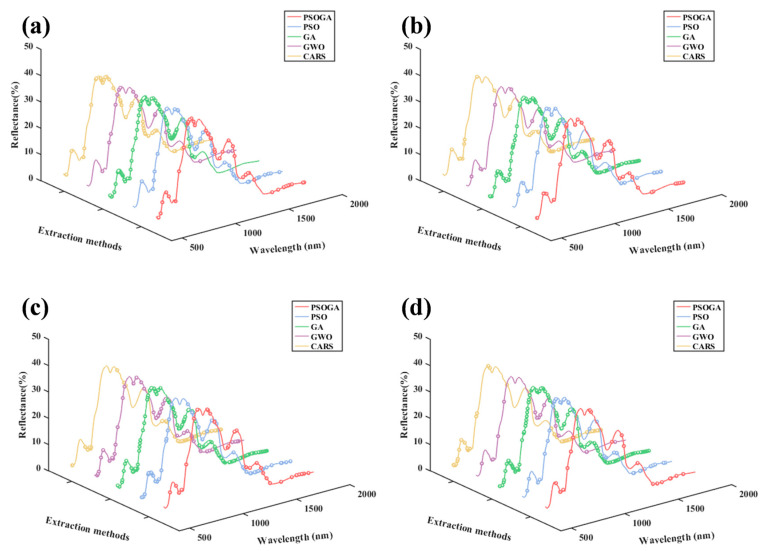
Comparison of different feature selection methods for predicting beef TVB-N content and L*a*b*: (**a**) TVB-N content, (**b**) L*, (**c**) a*, (**d**) b*. The solid lines represent the average reflectance spectrum of the beef samples, while the circular markers on each line indicate the specific spectral wavelengths selected by the corresponding algorithm.

The distribution of the features selected using the five methods is shown in Figure 4. A critical observation is that GA retained a relatively high proportion of wavelengths (35.3–51.3% of the full spectrum). This pattern suggests that, for high-dimensional Vis-NIR data with significant collinearity, GA tended toward broader spectral coverage [38]. In contrast, the other four methods (PSOGA, PSO, GWO, CARS) selected a more parsimonious subset of features while preserving chemically informative wavelengths critical for prediction accuracy.

#### 3.3.2. Performance Comparison of XGBoost Models at Selected Wavelengths

**Figure 5:**
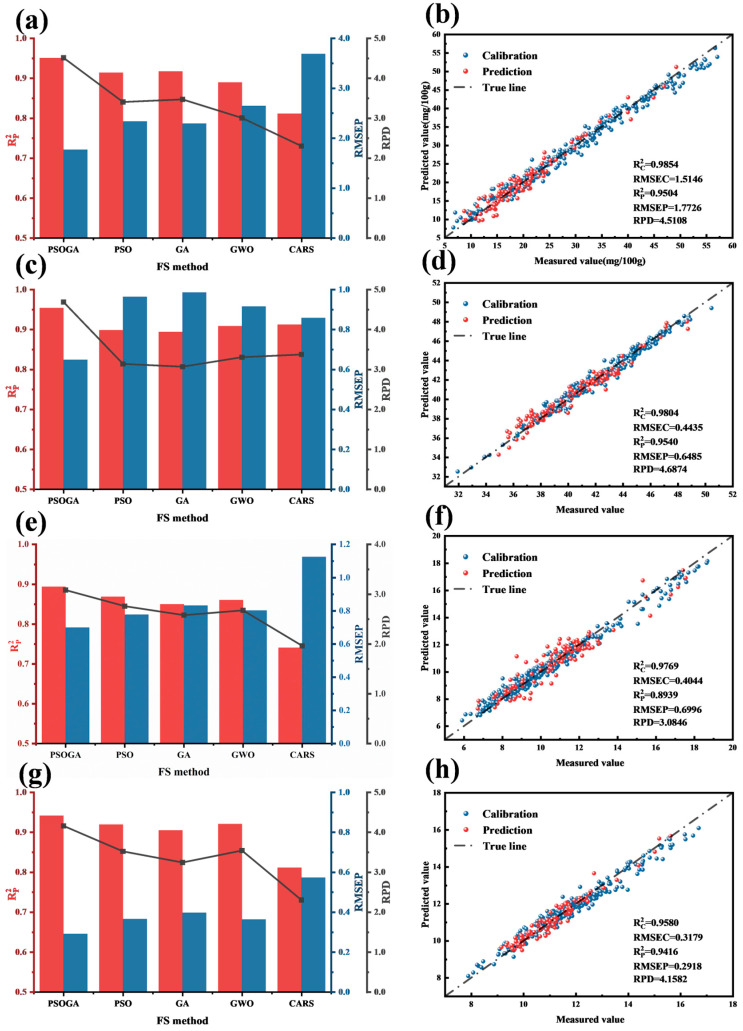
Comparison of the results of XGBoost prediction models built using different feature selection methods: (**a**) TVB-N content, (**c**) L*, (**e**) a*, (**g**) b*; scatter plots of actual versus predicted values for the calibration and prediction sets of the PSOGA-XGBoost prediction model for each metric: (**b**) TVB-N content, (**d**) L*, (**f**) a*, (**h**) b*.

The predictive performance of XGBoost models, trained on optimally preprocessed data (TVB-N, L*, a*, b*) and incorporating features selected by the five methods, is summarized in Figure 5 and Table 3. Feature selection significantly enhanced model performance. The baseline model (no feature selection) exhibited higher RMSEP and lower R^2^p and RPD values, indicating poor generalization and unreliable predictions. This underscores the necessity of feature selection to mitigate overfitting and improve interpretability.

Specifically, for TVB-N prediction, the PSOGA-XGBoost model attained an outstanding R^2^p of 0.9504 and an RMSEP of 1.7726 mg/100 g, representing a 14.2% increase in R^2^p and a 45.6% reduction in RMSEP compared to the full-spectrum model. Although PSOGA selected 41 features—more than GWO (19) but comparable to PSO (40)—it effectively balanced model parsimony with predictive power.

Regarding color parameters (L*, a*, b*), PSOGA similarly yielded the best results. For L*, the model achieved an R^2^p of 0.9540, significantly surpassing the baseline (R^2^p = 0.8301) and other methods (Rank: PSOGA > CARS > GWO > PSO > GA). For a* and b*, the PSOGA-XGBoost models achieved R^2^p values of 0.8939 and 0.9416, respectively, with notable improvements in RPD (3.0846 for a* and 4.1582 for b*). The substantial boost in RPD (>3.0 for a*, >4.0 for b*) confirms the robustness of the selected features. In contrast, while GA utilized the largest number of wavelengths, it did not yield the highest predictive accuracy among the tested algorithms, indicating that the inclusion of additional spectral variables did not necessarily translate to improved model generalization.

An RPD value exceeding 4, combined with the low RMSEP and high R^2^p, suggests that the PSOGA-XGBoost model possesses strong predictive capability. While RPD values above 4 generally indicate good screening potential, the narrow gap between RMSEC and RMSEP further corroborates the stability of the model and lack of overfitting. It is worth noting that, despite the high R^2^p values for TVB-N and L*, the model demonstrated strong robustness against overfitting. This is evidenced by the close proximity between the RMSEC and RMSEP values, as well as the consistent performance across the calibration and prediction sets. The slight gap between these metrics indicates that the PSOGA-XGBoost model has successfully captured the general biochemical patterns of beef freshness rather than fitting the random noise in the training data.

#### 3.3.3. Comparison of PSOGA with Other Evolutionary Algorithms

**Figure 6:**
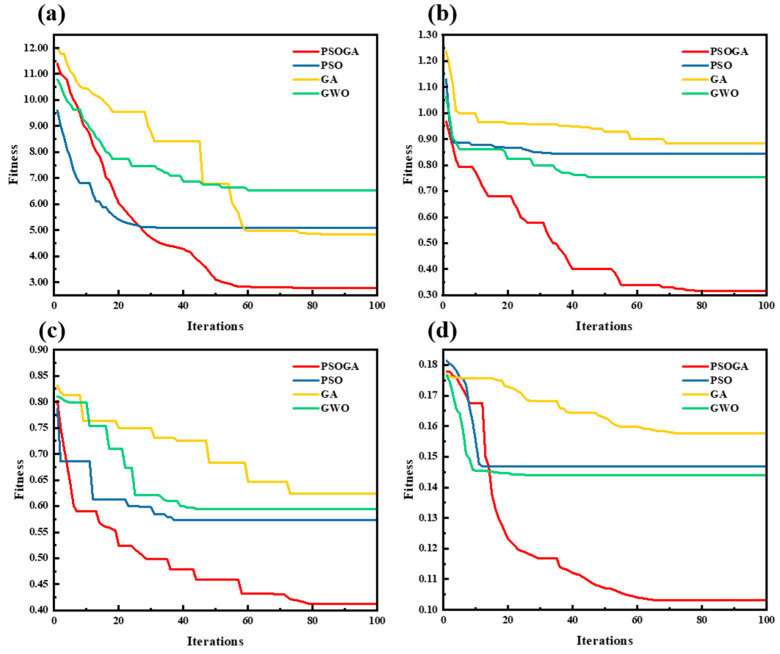
Iterative curves of fitness values of different algorithms on beef TVB-N content and L*a*b*: (**a**) TVB-N content, (**b**) L*, (**c**) a*, (**d**) b*.

In order to further explore the search efficiency and convergence characteristics of PSOGA, the iterative process of PSOGA is compared and analyzed with the intelligent optimization algorithms PSO, GA, and GWO, which also have explicit evolutionary mechanisms. The CARS method is not included in this part of the comparison because of its non-evolutionary variable screening mechanism [39], which does not have a comparable convergence process of the fitness function to ensure the scientificity and consistency of the analysis. The iterative optimization processes for TVB-N and color parameters are illustrated in Figure 6. While all four algorithms (PSO, GA, GWO, PSOGA) exhibited a downward trend in fitness over iterations, PSOGA consistently achieved the lowest final error values and faster convergence rates across all indicators. Unlike the standard PSO and GA, which showed tendencies to stagnate in local optima after approximately 20–30 iterations, the PSOGA maintained a continuous descent. This superior performance is attributed to the integration of the adaptive Lévy flight strategy and genetic operators, which effectively maintained population diversity and enabled the algorithm to escape local minima [40]. Consequently, PSOGA demonstrated notably improved stability and search efficiency compared to the single-mechanism algorithms.

### 3.4. Model Interpretation by SHAP Framework

**Figure 7:**
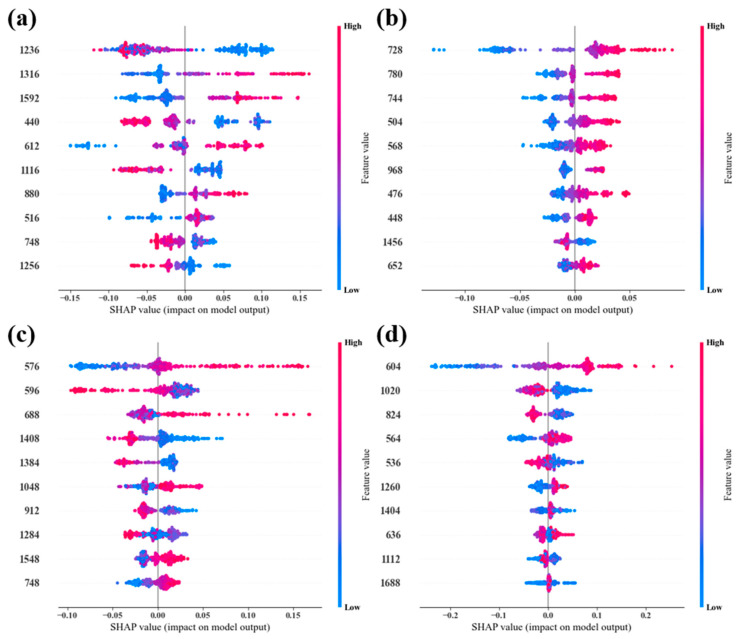
(**a**) TVB-N content, (**b**) L*, (**c**) a*, and (**d**) b* using PSOGA selected wavelengths for the best prediction model with SHAP value beeswarm plots.

In order to comprehensively comprehend the decision-making mechanism of the model and accurately quantify the contribution of each feature wavelength to the prediction outcomes, this research employed the SHAP framework to perform an interpretability analysis of the optimal PSOGA-XGBoost model. Figure 7 shows the SHAP beeswarm plots for the top 10 most important feature wavelengths in each indicator prediction model. Each point represents a sample, with point colors ranging from blue to red indicating the reflectance of the feature (at the wavelength) from low to high. The SHAP values on the x-axis indicate the magnitude and direction of the feature’s contribution to the model output.

As shown in Figure 7a, 1236 nm and 1316 nm emerged as the most significant features for TVB-N prediction. The high reflectance at 1236 nm (C–H second overtone) correlates with negative SHAP values, while 1316 nm and 1592 nm (N–H/O–H overtones) show positive contributions [33]. Additionally, the influence of the 440 nm band indicates that the accumulation of TVB-N is closely linked to moisture state changes alongside protein degradation and lipid oxidation [30]. These results confirm that the model accurately captures the synergistic biochemical shifts occurring during spoilage.

For the L* value (Figure 7b), 728 nm is the dominant feature, where higher reflectance positively shifts the predicted brightness. Other contributing wavelengths in the visible range (504–780 nm) align with the myoglobin states previously discussed in Section 3.1. Conversely, the negative impact of 1456 nm (O–H region) suggests that water migration affects light scattering and surface appearance [41]. Thus, the model effectively balances pigment oxidation and physical scattering effects to predict meat lightness.

In the a* model (Figure 7c), 576 nm exhibits a strong positive effect, directly correlating with the absorption peak of oxymyoglobin and the characteristic bright red color of fresh beef [42]. In contrast, the reflectance at 408 nm (Soret band) and NIR wavelengths (912 nm, 1284 nm) shows negative correlations, reflecting the transition toward metmyoglobin and protein denaturation [43]. These SHAP patterns validate that the model’s “redness” prediction is grounded in established myoglobin chemistry.

As illustrated in Figure 7d, the yellowing of beef (b*) is primarily governed by 604 nm and 564 nm, associated with the accumulation of metmyoglobin during the browning period [31]. Wavelengths related to water and organic compounds (1020 nm, 1404 nm) show negative correlations, potentially suppressing the yellow hue [44].

Through the detailed SHAP analysis described above, we not only quantified the contribution of each key wavelength to the model prediction, but more importantly, combined this spectral information with the biochemical composition and spoilage mechanisms of beef, greatly enhancing the transparency and credibility of the model [45]. This provides a solid theoretical foundation for the future development of more targeted non-destructive testing equipment and methods. Crucially, the alignment of these key wavelengths with specific chemical bonds confirms the physical validity of the high model accuracy reported. Unlike “black-box” models that might rely on spurious correlations to achieve high R^2^, the SHAP analysis validates that the PSOGA-XGBoost model relies on genuine biochemical signals associated with spoilage, thereby further mitigating concerns regarding potential overfitting.

### 3.5. Comparative Discussion with Recent Studies

To further evaluate the performance and positioning of the proposed framework, a comprehensive comparison was conducted with recent studies on meat quality assessment. In a previous study, Li et al. [46] utilized Hyperspectral Imaging combined with PLSR and LSSVM to predict beef freshness, achieving an R^2^p of 0.87–0.96. Similarly, Tang et al. [47] applied AdaBoost to monitor quality changes in frozen pork using Vis-NIR spectroscopy, demonstrating the potential of ensemble learning in meat analytics. However, most of these studies rely on traditional feature selection or manual band ascription, which, as noted by Cai et al. [48], often lacks a universal “golden combination” across different data sources, leading to time-consuming trial-and-error experiments to identify the optimal processing parameters.

In contrast, the PSOGA-XGBoost framework introduced in this research demonstrates superior efficiency and accuracy. By integrating Logistic chaotic mapping and Lévy flight strategies, the improved PSOGA automatically navigates the high-dimensional spectral space to identify the most discriminative wavelengths, effectively avoiding the premature convergence common in standard meta-heuristic algorithms. Our model achieved a quantitative prediction accuracy for beef TVB-N (R^2^p = 0.9504, RMSEP = 1.7726 mg/100 g), which is highly competitive with or superior to the results reported in the recent literature. Furthermore, while advanced deep learning models like the CNN-Bi-LSTM developed by Yan et al. [49] offer high predictive power (R^2^p ≈ 0.90), they are often criticized for their “black-box” nature, which obscures the underlying chemical relationship between spectral response and meat spoilage.

A key distinction of our study is the integration of the SHAP framework to decode the XGBoost model. Unlike the aforementioned studies that focus primarily on prediction metrics, our approach explicitly quantifies the contribution of each selected wavelength and links them to specific biochemical mechanisms, such as protein degradation (N–H bonds) and moisture migration (O–H bonds). This automated feature extraction combined with mechanistic interpretability significantly reduces the reliance on manual empirical design while improving the model’s transparency. In conclusion, these results demonstrate that the PSOGA-XGBoost-SHAP framework significantly outperforms traditional non-destructive methods in both precision and interpretability, providing a robust new tool for future research in food quality monitoring.

## 4. Conclusions

This study successfully developed a non-destructive beef freshness assessment method by integrating Vis-NIR spectroscopy with a novel PSOGA-XGBoost-SHAP framework. The results demonstrated that the PSOGA effectively reduced the high dimensionality of spectral data, selecting a compact subset of informative wavelengths that outperformed traditional feature selection methods in both convergence speed and predictive accuracy. The XGBoost models built on these selected features achieved a robust performance for predicting TVB-N and color parameters (L*, a*, b*), confirming the feasibility of the proposed approach for quality monitoring. Furthermore, the integration of SHAP analysis provided critical interpretability, revealing that the selected wavelengths were directly linked to biochemical changes such as myoglobin oxidation and water–protein interactions, thereby validating the model’s reliability from a mechanistic perspective.

Despite these promising results, this study has limitations. The current models were developed using beef samples obtained from a specific regional market, which may limit their immediate generalizability to diverse breeds or meat cuts from different geographic origins. Additionally, the spectral acquisition was performed offline in a static setting. Future research should focus on validating these models on independent datasets involving multiple beef varieties and on developing online Vis-NIR systems capable of real-time monitoring in industrial processing lines.

## Figures and Tables

**Table 1:** Boundary conditions for XGBoost hyperparameters.

Hyperparameter	Range
n_estimators	100–500
learning_rate	0.01–0.3
max_depth	3–8
gamma	0
reg_lambda	0
reg_alpha	1

**Table 2:** Statistics summary of measured TVB-N and L*a*b* values.

Content	Calibration (*n* = 315)		Prediction (*n* = 105)	
	Mean ± SD	Range	Mean ± SD	Range
TVB-N (mg/100 g)	28.44 ± 12.52	6.65–66.05	20.37 ± 7.96	8.66–49.27
L*	42.02 ± 3.17	31.94–50.46	40.37 ± 3.03	34.93–48.74
a*	10.48 ± 2.68	5.90–18.69	11.01 ± 2.04	6.08–18.03
b*	11.72 ± 1.54	7.88–16.70	11.18 ± 1.29	8.87–15.29

**Table 3:** Comparison of XGBoost model results based on different feature selection methods.

Content	Methods	R^2^c	RMSEC	R^2^p	RMSEP	RPD
TVB-N (RAW)	/	0.7656	6.0641	0.8322	3.2601	2.4409
	PSOGA	0.9854	1.5146	0.9504	1.7726	4.5108
	PSO	0.9332	3.2376	0.9137	2.3377	3.4041
	GA	0.9551	2.6543	0.9170	2.2931	3.4703
	GWO	0.9727	2.0702	0.8894	2.6468	3.0066
	CARS	0.9394	3.0612	0.8113	3.6882	2.3021
L* (SG)	/	0.8753	1.1190	0.8301	1.2469	2.4260
	PSOGA	0.9804	0.4435	0.9540	0.6485	4.6874
	PSO	0.9637	0.6039	0.8985	0.9637	3.1391
	GA	0.9750	0.5007	0.8938	0.9857	3.0690
	GWO	0.9538	0.6810	0.9086	0.9148	3.3068
	CARS	0.9030	1.0145	0.9121	0.8576	3.3736
a* (SG + 1D)	/	0.8814	0.9166	0.8193	0.9130	2.3523
	PSOGA	0.9769	0.4044	0.8939	0.6996	3.0846
	PSO	0.9677	0.4786	0.8689	0.7777	2.7617
	GA	0.9796	0.3798	0.8499	0.8320	2.5812
	GWO	0.9644	0.5023	0.8604	0.8023	2.6767
	CARS	0.9676	0.4769	0.7408	1.1250	1.9642
b* (RAW)	/	0.7958	0.6960	0.8941	0.4195	3.0733
	PSOGA	0.9580	0.3179	0.9416	0.2918	4.1582
	PSO	0.9270	0.4161	0.9193	0.3662	3.5199
	GA	0.9425	0.3694	0.9049	0.3976	3.2424
	GWO	0.9031	0.4795	0.9203	0.3639	3.5426
	CARS	0.9271	0.4106	0.8115	0.5735	2.3035

## Data Availability

The data presented in this study are available on request from the corresponding author.

## Supplementary Materials

The following supporting information can be downloaded at: https://www.mdpi.com/article/10.3390/foods15040728/s1, Table S1: Summary of key wavelengths identified by SHAP and their biochemical assignments. Reference [50] is cited in the supplementary materials.
