# Supplementary material for: Non-Destructive Assessment of Beef Freshness Using Visible and Near-Infrared Spectroscopy with Interpretable Machine Learning
Source: Foods. 2026 Feb 15;15(4):728. doi: 10.3390/foods15040728 (PMC12939979; doi:10.3390/foods15040728)
Supplement: Supplementary file 1 [file foods-15-00728-s001.zip › foods-4142536-supplementary.pdf]

S1. Logistic Mapping Initialization Population

In population intelligence optimization algorithms, the diversity of the initial population is crucial to the global search capability of the algorithm. In standard PSO algorithms, the initial positions of the particles are usually randomly generated, which may lead to uneven distribution of individual populations and thus affect the convergence accuracy of the algorithm. The basic idea of chaotic mapping is to make use of the ergodicity and randomness of chaotic variables to map the variables to be optimized into the value intervals of chaotic variables through chaotic mapping rules, and transform the obtained chaotic sequences into the search space of the objective function through linear transformation. Due to its characteristics of randomness, ergodicity and regularity, it is able to produce a more homogeneous sequence than a completely random distribution. Based on this, this study chooses to use Logistic chaotic mapping to initialize the particles in the population. In this study, Logistic chaotic mapping is used to generate the initial population. The distribution of the initial positions is more uniform compared to the overall population generated by the random initialization method.

Logistic mapping generates chaotic sequences by Eq. (S1):

$$x_{n+1} = r \cdot x_n \cdot (1 - x_n) \tag{S1}$$

Where $x(n)$ is the n th chaotic sequence value, r is the control parameter, $r \in (0,4)$, The parameter r is set to 4 in this study, as the system exhibits fully chaotic behavior within the range $x_n \in [0,1]$ only when $r = 4$, ensuring maximum randomness and ergodicity for population initialization.

S2. Elite Particle Segmentation

In PSO, each particle carries its current position information. At the beginning of each iteration, the fitness values of all particles are computed and then sorted in ascending order. The sorted population is subsequently divided into two distinct subgroups based on an adaptive classification coefficient, K: an elite subpopulation consisting of particles with superior fitness (specifically, those with an ordinal rank $i \leq K$), and an ordinary subpopulation comprising the remaining particles. To enhance the randomness of global search, a stochastic perturbation term is introduced into the calculation of K. The formula for computing K is Eq. (S2):

$$K = \mathrm{ceil}\left(\mathrm{rand} \times N \times \frac{1}{1 + e^{5T-10t}}\right) \tag{S2}$$

where ceil is the ceiling function, rand is a uniformly distributed random number in the range [0, 1], N is the population size, t is the current iteration number, and T is the maximum number of iterations.

The elite subpopulation, possessing superior fitness values, inherently contains more valuable

information; thus, to preserve their excellent characteristics, an improved inertia weight is incorporated into their velocity update formula. Conversely, ordinary particles retain a higher degree of search freedom to enhance global exploration capabilities. The inertia weight, ω, is a critical parameter that balances global exploration and local exploitation: a larger ω reduces reliance on the initial population and enhances the particles' ability to explore new regions, while a smaller ω promotes finer local search. This study proposes a non-linearly decreasing inertia weight strategy, leveraging the continuous, smooth, and strictly monotonic properties of the Sigmoid function. Consequently, the velocity update formula for the elite subpopulation is defined by Eq. (S3):

$$v_i^{t+1} = \omega^t \cdot v_i^t + c_1 r_1 \left(P_{\text{best},i} - x_i^t\right) + c_2 r_2 \left(G_{\text{best}} - x_i^t\right) \tag{S3}$$

where t represents the current iteration number, c_1 and c_2 are the individual and social learning factors, respectively, r_1 and r_2 are random numbers uniformly distributed within (0,1), and $P_{\text{best},i}$ and G_{best} represent the individual best position and the global best position, and ω^t is the non-linearly decreasing inertia weight, calculated by Eq. (S4):

$$\omega^t = \omega_{min} + \frac{\omega_{max} - \omega_{min}}{1 + e\left(-5\left(\frac{2t}{T} - 1\right)\right)} \tag{S4}$$

where ω_{max} and ω_{min} are the upper and lower limits of the inertia weight, respectively, t is the current iteration number, and T is the maximum number of iterations.

S3. Adaptive Lévy Flight Mutation

In traditional GA algorithms, mutation operations typically alter chromosome genes based on fixed probabilities or rules. This mechanism often leads to premature convergence in local optima, especially within complex search spaces. To address this, this study integrates Lévy flight into the mutation process. By leveraging its heavy-tailed distribution properties, Lévy flight facilitates long-distance random jumps, enabling the algorithm to break through local attraction basins and explore uncharted regions, thereby significantly enhancing the probability of discovering global optimal solutions. The step generation mechanism of Lévy flight is based on a stable distribution, and the step length is calculated by Eq. (S5):

$$L = \frac{u}{|v|^{1/\beta}} \tag{S5}$$

where $u \sim \mathcal{N}(0, {\sigma_u}^2), v \sim \mathcal{N}(0,1)$, indicating that u and v are random numbers drawn from a normal distribution with a mean of 0 and variance of σ_u^2, and a standard normal distribution, respectively. σ_u is a scale factor for the normal distribution related to β, calculated by Eq. (S6):

$$\sigma_u = \left[\frac{\Gamma(1+\beta)\sin\left(\frac{\pi\beta}{2}\right)}{\Gamma\left(\frac{1+\beta}{2}\right)\beta 2^{(\beta-1)/2}}\right]^{1/\beta} \tag{S6}$$

where $\beta \in (0,2)$, typically set to 1.5, and Γ is the standard Gamma function. This value was selected based on prior studies [1] as it offers a balanced trade-off between the heavy-tailed distribution required for long-distance jumps and the smaller steps needed for local refinement.

After selecting the gene locus x for mutation, the chromosome mutation formula is Eq. (S7):

$$x' = x + \eta \cdot L \cdot (x_{max} - x_{min}) \qquad (S7)$$

where x_{max} and x_{min} represent the upper and lower bounds of the gene value range, ensuring that the mutated solution remains within the feasible search space. $\eta \in (0,1]$ is an adaptive adjustment coefficient used to control the relative magnitude of the Lévy jump. It typically decreases linearly with increasing iterations, and it is calculated by Eq. (S8):

$$\eta(t) = \eta_{\max} - (\eta_{\max} - \eta_{\min}) \times \frac{t}{T} \qquad (S8)$$

where $\eta_{\max}$ and $\eta_{\min}$ are the upper and lower limits of η, respectively, t is the current iteration number, and T is the maximum number of iterations. This linear decreasing strategy allows η to maintain a larger value in the early stages of the algorithm, granting Lévy jumps a greater relative magnitude to promote global exploration and help the algorithm escape local optima. Conversely, in the later stages, η gradually decreases, restricting the magnitude of Lévy jumps and encouraging refined local exploitation, thereby preventing overly large step sizes from overshooting potential optimal regions.

This Lévy flight mutation mechanism, by introducing random long-distance jumps, significantly enhances the algorithm's probability of escaping local optima and, consequently, its global exploration capability.

[1] A. Qi, D. Zhao, F. Yu, A.A. Heidari, H. Chen, L. Xiao, Directional mutation and crossove r for immature performance of whale algorithm with application to engineering optimization, Jo urnal of Computational Design and Engineering, 9 (2022) 519–563.

Table S1 Summary of key wavelengths identified by SHAP and their biochemical assignments.

Indicator	Key Wavelengths (nm)	Biochemical Assignment
TVB-N	1236, 1316, 1592; 440	C-H, N-H; O-H stretching
$L*$	728, 780; 1456	Myoglobin forms; O-H
$a*$	576; 408	Oxymyoglobin; Soret band
$b*$	604	Metmyoglobin
